# Development and validation of the pesticides label (pictograms and color codes) questionnaire: A pilot study of farmers’ understanding and practices in Lebanon

**DOI:** 10.1371/journal.pone.0321591

**Published:** 2025-06-09

**Authors:** Mona Aridi, Sara Abou Ibrahim, Nisreen Alwan, Wissam Ghach

**Affiliations:** 1 LARIS, University of Angers, Angers 49000, France; 2 Faculty of Arts and Sciences, Department of Education, Modern University for Business and Science, Damour, Lebanon; 3 College of Health Sciences, Abu Dhabi University, Abu Dhabi, United Arab Emirates; 4 Department of Public Health, Canadian University Dubai, Dubai, United Arab Emirates; 5 The Global Health Network – Midde East and North Africa (TGHN – MENA), Dubai, United Arab Emirates; Hamadan University of Medical Sciences, IRAN, ISLAMIC REPUBLIC OF

## Abstract

Farmers’ comprehension of color codes and pictograms of pesticides is crucial for ensuring the safe and effective use of these agricultural chemicals. These visual aids provide essential information about hazards, proper handling, and application methods. This understanding helps prevent accidents, protect health, and promote sustainable farming practices. This study aims to develop and validate a Pesticide Label Questionnaire (PLQ) qualitatively and quantitatively using psychometric measures to evaluate farmers’ comprehension of pictograms and color codes as well as the technical sign-related practices in Lebanon. The questionnaire, consisting of 29 statements, was generated and developed by experts in the field of agriculture, chemistry, environmental health, public health, and statistics. Face, content, and construct validity were assessed, and psychometric properties were examined for data validation. Internal consistency and reliability were measured using Cronbach’s alpha (α) and Guttman’s Lambda Coefficient. Statistical analyses were performed using Statistical Package for the Social Sciences (SPSS version 27) and RStudio (Version 1.1.456, Inc., 2009–2018). The Construct Validity analyses demonstrated strong reliability and coherence within the questionnaire, with an overall Cronbach’s alpha of 0.873. Section I (“Pictorial and Color Code Comprehension”) achieved a Cronbach’s alpha of 0.849, while section II (“Technical Sign-related Practice Assessment”) showed moderate reliability at 0.667. Factor analyses revealed a well-defined structure, with five components explaining 62.03% of the variance in section I and three components accounting for 61.81% in section II. These results highlight the questionnaire’s robust construct validity and its ability to capture key dimensions of comprehension and practice related to pesticide labels. The results of this study indicate that the 29-item PLQ is a valid and reliable tool for evaluating farmers’ understanding of pictograms and color codes along with technical sign-related practices.

## 1. Introduction

Agriculture is one of the largest sources of livelihood worldwide [1]. Due to the increase in population and the demand for high-quality and superior food products, recent reports have highlighted the increase in the global use of pesticides in the agriculture sector [2]. Despite their protective role and their contribution to increased crop yield, pesticides may have adverse effects on the environment and public health through direct exposure of end-users to pesticides or through public exposure to residues in the food chain and environmental resources especially when farmers do not handle pesticides safely [3,4]. Therefore, it is imperative to prioritize safe pesticide handling practices and implement sustainable agricultural strategies to mitigate the adverse health and environmental impacts associated with pesticide use.

According to World Health Organization (WHO) reports, pesticides are among the leading causes of death by self-poisoning among end-users, and this issue is disproportional in low- and middle-income countries where agricultural workers are at higher risk of the adverse health effects of pesticides [5]. Additionally, the Food and Agriculture Organization (FAO) suggests that 70% of pesticides used in developing nations could be lost to the environment due to inadequate application methods [6]. Such loss contributes to drift, volatilization, deposition in soil, and nature such as runoff from rain [7]. While alternative methods such as targeted drone spraying have shown greater efficiency; limited access and knowledge about these technologies among the farming community in developing countries hinder their widespread adoption [8]. To enhance safe pesticide usage, label information, including FAO pictograms and WHO color codes, are recommended to provide technical recommendations as well as safety instructions for risk communications with end users [9]. In many countries, these labels are enforceable by law and should be part of the pesticide package to guarantee that pesticide applications comply with international guidelines and local regulations [9–12]. However, studies indicate that only a small percentage of end-users can read and understand the technical information on the pesticide labels and the significant proportion of farmers struggle to interpret these labels due to limited literacy, lack of training, and inadequate awareness [10,11].

Literature studies have highlighted a varied effectiveness of label pictograms and color codes, among their farming communities, where the lack of knowledge and labels comprehension as well as lack of proper training seem to be a major cause of the inappropriate use of pesticides [10,11,13–18]. Thereby, assessing the farmers’ understanding of the pesticide label, especially pictorial and colored messages through a well-designed measurable questionnaire is crucial and essential to address the weaknesses of pesticide labels as a risk communication tool and track the incompetencies of the farmers’ knowledge and skills in reading, analyzing, and understanding the correct meaning of FAO pictograms and WHO color codes which are displayed on the pesticide label.

Farmers’ understanding of pesticide labels (especially FAO pictograms and WHO color codes) plays a crucial role in encouraging safe and conscientious pesticide application practices in developing countries such as Lebanon [19]. In fact, the Lebanese agricultural sector rely heavily on agrochemicals which comprise pesticides and fertilizers for crop protection [20], thus exposing humans and the environment to the harmful effects of pesticides when pesticides are not handled properly [21–24]. Despite these alarming expectations, a single study was conducted to evaluate farmers’ understanding of these pictorial and colored messages to show that 8 out of the 14 FAO pictograms and 1 out of the 4 WHO color codes are correctly perceived by at least 50% of the farmers [13]. This study underscored the need for further investigations using a validated tool to generalize these findings in Lebanon and similar developing countries [13]. For the first time, the current study aims to pilot the creation and development of a reliable and psychometrically validated tool to measure the farmers’ understanding of FAO pictograms and WHO color codes on pesticide labels. Using psychometric measures, technical sign-related practice items will evaluate whether the subsequent consequences of farmers’ misuse can be concluded from the poor comprehension of FAO pictograms and WHO color codes. This paper aims to validate the instrument qualitatively and quantitatively using psychometric measures to be applied in both developed and developing countries. Through this tool, a better understanding of the farmers’ strengths in both theoretical and practical knowledge will be provided to highlight the necessity of designing training programs on the technical signs of pesticides as part of the United Nations (UN) sustainability goals of well-being (Sustainable Development Goal 3, SDG 3), quality education (Sustainable Development Goal 4, SDG 4), and life on land (Sustainable Development Goal 15, SDG 15).

## 2. Materials and methods

### 2.1. Study design

**Fig 1:**
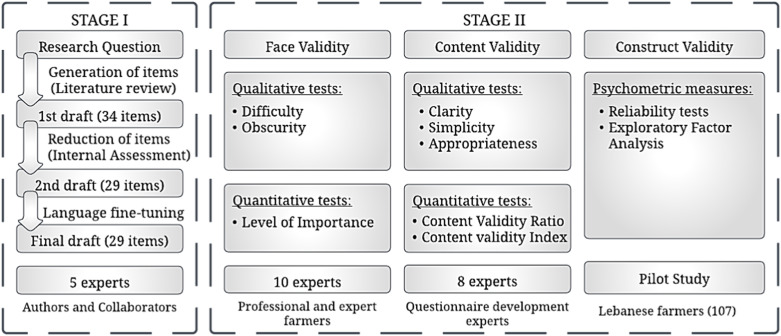
An overview of the validation process.

A comprehensive assessment model incorporating both qualitative and quantitative methods was utilized to ensure the rigor and validity of the developed questionnaire. The questionnaire underwent two distinct stages: Stage I, involved item generation and development, while Stage II, focused on instrument testing and validation. Fig 1 depicts the validation process undertaken in this study, beginning with the research question, and progressing through three iterative drafts, culminating in the final step of construct validity, after passing through face validity and content validity assessments.

#### Stage I—Qualitative phase: (Development of the pesticides questionnaire).

This stage included two steps: [1] Generation of the items; and [2] Item reduction and development of the PLQ.

**Generation of the Items:** The need for an effective assessment tool was generated from the ongoing acute pesticide poisoning reported each year worldwide [5] and the literature recommendation of the effectiveness studies about pesticide labels especially pictograms and color codes [10,11,13]. To address this, the core structure of the PLQ was built according to the FAO Guidance on Good Labelling Practice for Pesticides [25]. The initial version of PLQ comprised 34 items, carefully constructed to ensure clarity, readability, and comprehension using straightforward language. The items were phrased both positively and negatively to prevent one-directional responses. In response to the recommendations of the literature studies, the foundation of the assessment tool included two main sections: the “Pictorial and Color Code Comprehension Assessment” (22 items) and the “Technical Sign-related Practice Assessment” (12 items) to encompass every aspect pertinent to the study’s objectives.

**Item Reduction and Development of the PLQ:** In the initial draft, a panel of five experts representing backgrounds in chemistry, agricultural engineering, public health, environmental health, and statistics, approved the 34-item questionnaire development. Criteria for item acceptance to the subsequent phase included suitability for the factor under examination, relevance to farmers’ understanding, and clarity of language. Items were either retained, revised, or eliminated based on the experts’ evaluation criteria. Five items did not meet the assessment criteria and were unanimously recommended for removal from the prospective questionnaire by all experts, leaving 29 items only. However, modifications were made to 7 items to better align them with the questionnaire’s objectives. The revised and retained items constituted the second draft of the questionnaire. Before proceeding to the next phase of validation, the questionnaire underwent translation from English to Arabic to ensure clarity and eliminate language barriers for farmers. To facilitate understanding, both English and Arabic versions were provided in a single document, ensuring accessibility and comprehension of each item.

Concerning the qualitative evaluation conducted by the five experts, certain items underwent slight adjustments to enhance their clarity and explicitness. Following the face and content validation process and subsequent evaluation by the experts, no additional modifications were deemed necessary.

A 29-item questionnaire of two sections was developed (Appendix A in S1 File). In the first section (Pictorial and Color Code Comprehension Assessment), 19 binary items addressed the comprehension of the pictorial and colored messages on pesticide labels such as FAO pictograms (Items 1–14), WHO color codes (Items 15–18), and one item (Number 19) merging some FAO pictograms and a WHO color code in the form of a sequence (as represented on the pesticide product label). In the second section (Technical Sign-related Practice Assessment), ten Yes-No items addressed farmers’ compliance with label-related practices (*e.g.*, PPE use, environmental protection, storage condition, and body hygiene) with the availability of stating the reason behind the negative responses (Items 20–29).

#### Stage II—Quantitative phase/Psychometric properties of the PLQ.

This stage included three steps: [1] Face validity; [2] Content validity; and [3] Construct validity.

**Face Validity:** Face validity was assessed to determine whether an indicator appears to be a sensible measure and fair representation of its underlying construct. This was accomplished through a combination of qualitative and quantitative methods. Qualitative face validity was gauged using a questionnaire (Appendix B in S1 File), where 10 qualified expert farmers were asked to rate the difficulty and clarity of each item on a 4-point Likert scale. These farmers included individuals with varying levels of education: five had elementary education, three had vocational certificates, and two were illiterate. Additionally, quantitative face validity was evaluated by the same group of farmers using another questionnaire (Appendix B in S1 File), where they determined the importance of each item using a 4-point Likert scale and calculated an impact score for each item.

The face validity findings regarding the difficulty, clarity, and significance of each item were assessed by 10 experts using a 4-point Likert scale. For difficulty, ratings ranged from 1 (very difficult) to 4 (not difficult); for obscurity, from 1 (very obscure) to 4 (not obscure); and for importance, from 1 (not relevant) to 4 (highly relevant). These ratings were then averaged by dividing the sum of the experts’ ratings by 40, which represents the sum of the maximum possible ratings provided by the 10 experts.

**Content Validity:** Content validity pertains to the degree to which an instrument comprehensively measures a particular construct across all its facets and dimensions, assessed by scrutinizing the validity of inferences drawn from test scores [26]. Both qualitative and quantitative techniques were employed to ascertain content validity. The qualitative evaluation involved consulting eight experts in tool development regarding the simplicity, clarity, and appropriateness of items within the questionnaire, considering their placement within it.

The evaluations provided by experts in the field of tool development were documented for each item. Qualitatively, each expert assessed the simplicity, clarity, and appropriateness of each item on a scale from 1 to 4 (with 1 representing the minimum and 4 representing the maximum). To calculate the ratio for each item, the total votes from the eight experts were divided by 32 (the sum of the maximum ratings provided by the eight experts).

The quantitative assessment involved analyzing the Content Validity Ratio (CVR) and the Content Validity Index (CVI). A panel of experts was contacted via telephone, and the questionnaire was presented to them to rate the necessity and relevance of each item.

*Content validity ratio*: At this stage, Lawshe’s model [31] was utilized to determine the CVR for the questionnaire. Experts assessed the necessity of each item, categorizing their feedback as essential, useful, not essential, or not useful. The experts’ evaluations were quantified using the CVR: ${CVR}_{i}=\frac{N_{e}-\frac{N}{2}}{\frac{N}{2}}$, where *CVR*_*i*_ represents the CVR value for the i^th^ measurement item, N_e_ is the number of panelists indicating a measurement item as “essential,” and N is the total number of panelists.

According to the content validity equation, the ratios can range from -1 (Perfect disagreement) to +1 (Perfect agreement) [27]. As per Lawshe’s model, the acceptable CVR is contingent on the number of experts involved in the validation process [27]. In this study, the questionnaire was evaluated by eight experts, necessitating a critical cut-off value of 0.75. Items scoring below this critical value should be considered for removal.

*Content validity index*: The Content Validity Index (CVI) is widely employed to ensure the comprehensive validation of an instrument utilizing a 4-point Likert scale (1 = not relevant, 2 = somewhat relevant, 3 = relevant, 4 = very relevant). This index facilitates validation both at the item level (I-CVI) and at the scale level (S-CVI) by measuring the agreement ratio regarding the relevance of each item [28].

The I-CVI is calculated by dividing the number of experts who rates each item as “very relevant” by the total number of experts. CVIs are expected to fall within the range of 0.00 to 1.00, where items with CVI > 0.79 are deemed relevant, those between 0.70 and 0.79 require revision, and those with CVI < 0.70 are candidates for elimination [29].

The S-CVI was assessed using two methods: Universal Agreement among experts (S-CVI/UA) and Average CVI (S-CVI/Ave). S-CVI/UA is calculated by dividing the number of items with a CVI of 1.00 by the total number of items being examined. Instruments with UA exceeding 0.80 are classified as having high content validity. S-CVI/Ave is determined by dividing the sum of the I-CVIs by the total number of items being studied [30]. A questionnaire with an average value surpassing 0.90 demonstrates an excellent content validity.

**Construct Validity:** To establish the construct validity of the questionnaire, reliability testing was first conducted to evaluate the internal consistency of the items in each section. Cronbach’s Alpha and Guttman’s Lambda were calculated for sections I and II separately to ensure that items within each section measured the same underlying construct. Both measures demonstrated satisfactory levels of internal consistency, confirming the reliability of the questionnaire.

Exploratory Factor Analysis (EFA) was conducted to identify latent factors underlying the binary items. Given the binary nature of the data, a tetrachoric correlation matrix was used to capture accurate relationships between items. Factor extraction was performed using Principal Axis Factoring, and Promax rotation was applied to enhance the clarity and interpretability of the emerging factor structure. The EFA results provided a refined understanding of the factors and the relationships among the items.

**Fig 2:**
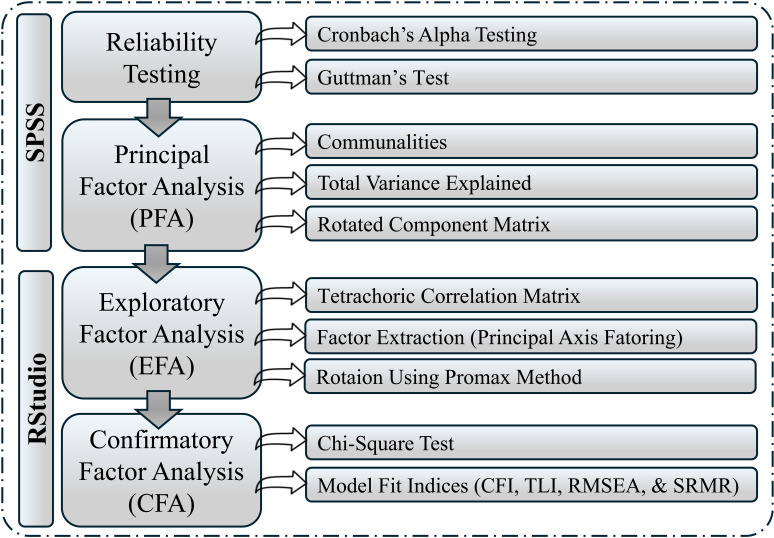
Outline for the construct validity process.

Finally, Confirmatory Factor Analysis (CFA) was conducted to test the hypothesized factor structure derived from EFA and confirmed its fit to the data. The CFA, performed using RStudio, involved evaluating multiple model fit indices, including the Comparative Fit Index (CFI), Root Mean Square Error of Approximation (RMSEA), and the Chi-Square Test. The analysis demonstrated a good fit between the model and the data, providing robust support for the construct validity of the questionnaire. An outline of the construct validation process is demonstrated in Fig 2.

The construct validity was carried out with the means of a pilot study of sample size of 107 farmers. Farmers from diverse backgrounds, ages, and experience levels were invited to participate in completing the questionnaire. While the estimated time for completion was approximately 15 minutes, some respondents took an average of 25 minutes to fill in all the required items due to their slower writing pace. Participants were instructed to complete the questionnaire without skipping any item.

### 2.2. Data collection

The pilot study was conducted between June 2022 and March 2023 on 107 farmers in three different agricultural regions in Lebanon (Mount Lebanon, Beqaa, and South Lebanon). The questionnaire was conducted during a seminar on the importance of pesticide precautions and awareness that was held in each of the three regions. Participation in the questionnaire was completely voluntary and anonymous. The rate of responsiveness reached 72.3%, 68.8%, and 66.7% in the three regions, respectively. Despite the good participation of the farmers in filling the questionnaire and the good level of responsiveness (66.7% - 72.3%), some of the farmers did not participate for reasons related to literacy, privacy concerns, or even paperwork issues, especially that among the audience were many illiterate and old farmers (aged above 60). “Convenience Sampling” describes our sampling technique, as the farmers who were readily available to provide us with their responses were only considered in this study.

### 2.3. Data analyses

The questionnaire’s reliability was evaluated through Cronbach’s Alpha and Guttman’s test. Furthermore, EFA, and CFA were carried out to approve the construct validity of the items. All these tests were investigated using the Statistical Package for the Social Sciences (SPSS Version 27 from IBM, Chicago, IL, USA) and RStudio (Version 1.1.456, Inc., 2009–2018). P-values less than 0.05 were considered significant.

### 2.4. Ethical considerations

The study protocol was approved by the Institutional Review Board (MU- 20220203–29) at the Modern University for Business and Science and was performed in accordance with the Declaration of Helsinki. In the first page, an informed consent was utilized to inform the participants about the aim and benefits of the study, the confidentiality, and the absence of potential risks. Completing the questionnaire filled by participants indicated their consent to be part of this study and that their participation was voluntary.

## 3. Results and discussion

According to our knowledge, this questionnaire represents a pioneering effort in Lebanon aimed at assessing farmers’ skills in reading and analyzing pesticide pictograms and color codes, offering valuable insights into their comprehension and technical sign-related practices according to FAO guidelines in the farming community in Lebanon and other developing countries.

### 3.1. Demographic data

All demographic data are summarized in Table 1. Among the 107 farmers who participated in the pilot study, there were 96 males (89.7%) and 11 females (10.3%). As for the age, there were three age groups classified as follows: 14.0% of the farmers belonged to the first age group [20, 39], 60.7% belonged to the second age group [40, 59], and 25.3% were aged 60 and above. Concerning the province, the study reached farmers in three main agricultural areas in Lebanon: Mount Lebanon (57.9%), Beqaa (30.8%), and South Lebanon (11.2%). As for the level of education, 20.6% of the farmers were illiterate, 61.7% had their primary-middle schooling, 7.5% received high school education or equivalent, and 10.3% were graduate or undergraduate college students. The survey was administered to 107 farmers across three provinces in Lebanon. Farmers were encouraged to provide feedback on any item they found unclear or expressions they found ambiguous. However, the feedback from farmers highlighted the clarity of the questionnaire language. The items were deemed clear, readable, and explicit in evaluating the farmers’ comprehension of their practices and the pictograms they utilize.

**Table 1:** Descriptive measures for the pilot study.

Variables		Frequency (n)	Percentage (%)
Gender	Male	96	89.7
	Female	11	10.3
Age	20–39	15	14.0
	40–59	65	60.7
	60 +	27	25.3
Province	Mount Lebanon	62	57.9
	Beqaa	33	30.8
	South Lebanon	12	11.2
Educational Level	Illiterate	22	20.6
	Elementary to middle school level	66	61.6
	High school level or equivalent	8	7.5
	University undergraduate level	8	7.5
	University graduate level	3	2.8
Pesticides’ Training	Yes	8	7.5
	No	99	92.5

### 3.2. Face validity

Examining the outcomes of the two qualitative aspects of face validity, namely the difficulty and obscurity of each item, the evaluations from the 10 experts varied between a minimum score of 0.88 and a maximum of 1.00 (Fig 2). As the validity scores are represented in terms of ratios that can be easily compared to 1, the lowest score of 0.88, recorded by items 19 and 23, is considered a high score and hence the level of agreement among the participating farmers is very good.

### 3.3. Content validity

After assessing the three qualitative aspects of content validity, including simplicity, clarity, and appropriateness of each item, the ratings provided by the eight experts ranged from a minimum of 0.81 to a maximum of 1.00, as shown in Fig 2. The lowest qualitative score in the content validity was 0.81 dented by questions 5, 12, and 19, which reveals a high level of agreement among the participating farmers.

#### 3.3.1. Content validity ratio.

The CVR values were computed for each item. Following Lawshe’s model, items evaluated by the eight experts with CVR below 0.75 were deemed non-essential and recommended for removal. None of the 29 items fell below this threshold, indicating that none should be eliminated. Furthermore, the average CVR for the entire questionnaire was 0.98, signifying a highly satisfactory CVR (Appendix C in S1 File).

#### 3.3.2. Content validity index.

The I-CVIs ranged from 0.88 to 1.00, and none of the tested items scored below the threshold (0.75). Consequently, none of the items required any revision or modification (Fig 2).

The Universal Agreement (UA) was calculated by dividing the number of items with a CVI of 1.00 (25 items) by the total number of items under examination (29 items): S-CVI/UA = 0.86. Additionally, the average was computed by dividing the sum of CVIs by 29: S-CVI/Ave = 0.98. Both the UA and the average approaches demonstrated high content validity, with S-CVI/UA > 0.80 and S-CVI/Ave > 0.90 (Appendix C in S1 File). Items 14, 19, 22, and 27 attained the lowest score (0.88) as compared to the score of other items; however, this score shows a high level of agreement between the farmers in the studied questionnaire.

**Fig 3:**
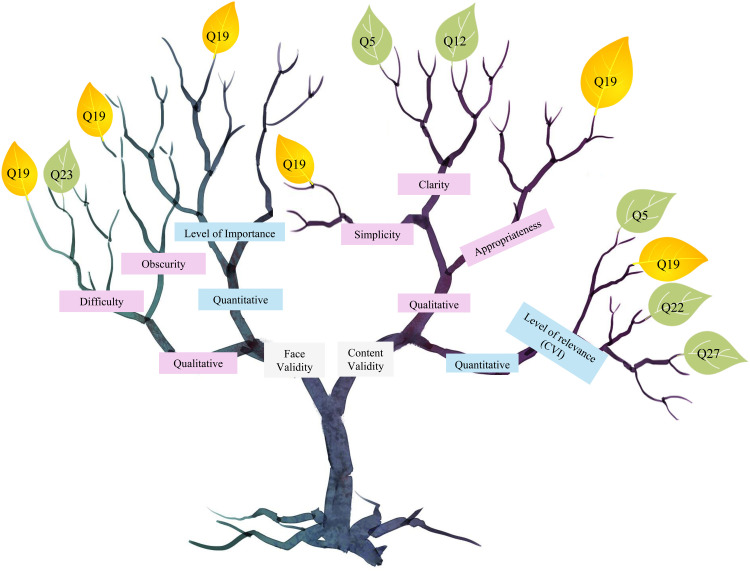
Tree diagram showing face and content validity - Items with the lowest scores.

The tree diagram in Fig 3 visually summarizes the items from the PLQ that received the lowest scores across various validity assessments, including face validity, content validity, and clarity. Question 19 (Q19) is prominently highlighted, indicating significant issues in terms of simplicity and clarity, as well as being perceived as obscure and difficult by participants. Additionally, Q19 appears repeatedly as a concern across multiple validity dimensions, suggesting it may not align well with the questionnaire’s objectives. Questions Q5, Q22, and Q27 are also noted for low scores compared to other items but they can still contribute to the aim of the questionnaire as long as the low score of each of them was reported once. This is contrary to Q19 as it needs further revision or clarification. This analysis underscores the necessity of refining Q19 to enhance the overall validity of the PLQ.

### 3.4. Construct validity

#### 3.4.1. Internal consistency and reliability.

To assess how consistent subjects’ responses remained over time, data for internal consistency across all questionnaire items were analyzed using the Cronbach alpha test (α = 0.873), revealing strong coherence among the items studied in the questionnaire. When examining internal consistency within each section of the questionnaire (Table 2), the findings indicated satisfactory reliability for both the “**Pictorial and Color Code Comprehension Assessment**” and “**Technical Sign-related Practice Assessment**” sections, with α = 0.849 and α = 0.667, respectively. Furthermore, Guttman’s Lambda coefficients were obtained to confirm the reliability of the results. The Lambda coefficients in the first section have shown very good reliability levels with Lambda-6 being the highest (0.894), indicating strong reliability confirming that the first section is highly reliable and robust. Similarly, the Lambda coefficients in section II indicated moderate to good reliability with Lamba-6 being the highest (0.723) suggesting acceptable reliability [31].

**Table 2:** Reliability test results for each section: Cronbach Alpha and Guttman Lambda coefficients.

Section I: Pictorial and Color Code Comprehension Assessment		Section II: Technical Sign-related Practice Assessment	
**Reliability Test**			
Cronbach’s Alpha	0.849	Cronbach’s Alpha	0.667
Number of items	19	Number of items	10
**Guttman’s Test**			
Lambda-1	0.804	Lambda-1	0.600
Lambda-2	0.859	Lambda-2	0.692
Lambda-3	0.849	Lambda-3	0.667
Lambda-4	0.817	Lambda-4	0.463
Lambda-5	0.838	Lambda-5	0.674
Lambda-6	0.894	Lambda-6	0.723

### 3.5. Exploratory factor analysis

#### 3.5.1. Tetrachoric correlation.

**Section I**

The tetrachoric correlation matrix for section I (Appendix D in S1 File) revealed key relationships between the 19 questions (Q1–Q19). Strong correlations (above 0.4) suggest that certain questions assess similar aspects of pictogram knowledge. For example, Q1 and Q2 (0.645) are closely related, likely testing basic pictogram understanding, while Q3 and Q6 (0.534) focus on hazard-related symbols. Moderate correlations, like Q14 & Q16 (0.432) and Q16 & Q18 (0.425), imply shared themes, possibly related to safety or specific pictogram attributes.

Weaker correlations (0.2–0.4) for pairs such as Q3 & Q4 (0.248) and Q8 & Q10 (0.212) suggest that these questions may address different or less related topics. Negative or negligible correlations, such as Q5 & Q6 (-0.007), indicate that some questions assess distinct knowledge areas. Additionally, the low standard errors suggest reliable estimates.

These results suggest that certain question pairs form clusters of related knowledge, while others highlight knowledge gaps or distinct areas of understanding. To improve knowledge assessment, it may be helpful to reinforce the key clusters (*e.g.,* Q1 & Q2, Q3 & Q6) in educational materials, while refining or providing additional support for questions with low correlations (*e.g.,* Q5).

**Section II**

The results of the tetrachoric correlation from section II, Technical Sign-related Practice Assessment, provided insights into the relationships between binary items (Appendix E in S1 File). The correlation matrix shows strong positive correlations, such as between Q27 and Q28 (0.6599), indicating a significant relationship. A moderate correlation was found between Q25 and Q24 (0.4861), while weaker or no associations were observed for pairs like Q20 and Q21 (-0.04188).

The standard error matrix shows moderate uncertainty in most correlation estimates, with standard errors typically ranging from 0.05 to 0.1. For example, the standard error for Q20-Q21 is 0.09677, indicating moderate confidence in the correlation values.

In summary, the correlation matrix identifies strong associations, and the standard error matrix supports the reliability of the correlations.

#### 3.5.2. Factor extraction.

**Section I**

The factor analysis for section I, focusing on Pictorial and Color Code Comprehension, identified three factors using Principal Axis Factoring with Promax rotation (Table 3). These factors, PA1, PA3, and PA2, explain 46% of the total variance. PA1, PA3, and PA2 contribute 16%, 15%, and 14%, respectively, to the variance. Factor loadings showed that PA1 is associated with items like Q2, Q4, Q7, Q15, and Q18, PA3 with Q5, Q10, Q12, and Q17, and PA2 with a mix of both positive and negative items, such as Q3 and Q13.

**Table 3:** Principal axis factoring with Promax rotation for section I.

	PA1	PA3	PA2
SS loadings	3.13	2.86	2.66
Proportion variance	0.16	0.15	0.14
Cumulative variance	0.16	0.32	0.46
Proportion explained	0.36	0.33	0.31
Cumulative proportion	0.36	0.69	1.00
Correlation of (regression) scores with factors	0.97	0.95	0.93
Mean item complexity			1.7
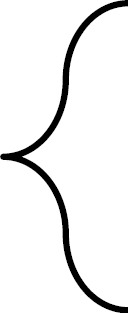 Model Fit Statistics	Root Mean Square of the Residuals (RMSR)		0.12
	Corrected RMSR		0.15
	Fit based upon off-diagonal values		0.81

The communalities ranged from 0.14 to 0.99, with Q2 and Q4 showing strong loadings. The average item complexity of 1.7 indicates that most items load on more than one factor. Factor correlations were low, suggesting that each factor represents a distinct construct. The model fit was good, with an RMSR of 0.12 and a corrected RMSR of 0.15. The fit based on off-diagonal values was 0.81, and the factor score correlations were high (PA1: 0.97, PA3: 0.95, PA2: 0.93).

Overall, the factor analysis supports a well-fitting model with three distinct factors, explaining a substantial portion of the variance. The factors represent different constructs, and the model adequately fits the data.

**Section II**

The factor analysis of section II, using principal axis factoring with a two-factor solution and Promax rotation, revealed two distinct factors (Table 4). Factor PA1, influenced by items Q27, Q28, and Q29, explains 41% of the variance, while Factor PA2, driven by items Q22, Q23, and Q26, accounts for 17%. Together, the two factors explain 58% of the total variance, indicating a good representation of the data. The correlation between PA1 and PA2 is 0.30, suggesting that they are related but distinct constructs.

**Table 4:** Principal axis factoring with Promax rotation for section II.

	PA1	PA2
SS loadings	4.13	1.67
Proportion variance	0.41	0.17
Cumulative variance	0.41	0.58
Proportion explained	0.71	0.29
Cumulative proportion	0.71	1.00
Correlation of (regression) scores with factors	0.98	0.95
Mean item complexity		1.2
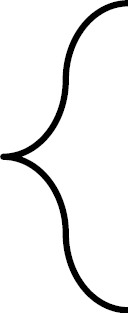 Model Fit Statistics	Root Mean Square of the Residuals (RMSR)	0.12
	Corrected RMSR	0.16
	Fit based upon off-diagonal values	0.93

The model fit is strong, with an RMSR of 0.12 and a corrected RMSR of 0.16, both within acceptable ranges. The fit based on off-diagonal values is excellent (0.93), and the correlations between factor scores and the factors themselves are high (PA1: 0.98, PA2: 0.95), confirming that the factors explain the variance well. Overall, the two-factor solution provides a meaningful and well-fitting representation of the data.

#### 3.5.3. Confirmatory factor analysis.

**Section I**

The CFA for section I, “Pictorial and Color Code Comprehension,” revealed mixed fit indices (Table 5). The Comparative Fit Index (CFI) is 0.892, indicating an acceptable fit, while the Tucker-Lewis Index (TLI) is 0.878, slightly below the ideal threshold. The Root Mean Square Error of Approximation (RMSEA) is 0.096, marginally above the acceptable range (0.05–0.08), and the Standardized Root Mean Square Residual (SRMR) is 0.115, indicating a slight misfit.

**Table 5:** The CFA for section I.

Model Fit Indices	CFI	0.892
TLI	0.878
RMSEA	0.096
SRMR	0.115

Factor loadings for Factor 1 (Q1–Q10) range from 0.218 to 1.360, with Q5 having the highest loading (1.360), while Factor 2 (Q11–Q19) loadings range from 0.249 to 2.067, with Q15 (2.067) being the strongest indicator. The covariance between the two factors is significant (0.115), suggesting a weak positive correlation. All item variances are significant, indicating that the model explains the variability in responses. Factor variances are also significant, with Factor 1 (0.173) and Factor 2 (0.083) confirming the meaningfulness of the latent constructs (Appendix F in S1 File).

The model uses the Diagonally Weighted Least Squares (DWLS) estimator, suitable for ordinal data. With 39 parameters, the model complexity is noted.

In conclusion, while the model demonstrated significant loadings and variances, the fit indices suggest room for improvement, particularly in RMSEA and CFI. Model revisions or modifications may be required to enhance fit.

**Section II**

The CFA for section II, “Technical Sign-related Practice Assessment,” indicates a generally good model fit with some areas for improvement (Table 6). The CFI (0.977) and TLI (0.969) indicate good fit, while the RMSEA (0.082) and SRMR (0.169) suggest minor misfit. Factor loadings are significant for most items, with Q21 having weak, non-significant loading, close to the boundary (0.3). The covariance between the two factors is moderate (0.189). Thresholds for most items are significant, and variances indicate acceptable reliability, especially for Q29 (Appendix G in S1 File).

**Table 6:** The CFA for section II.

Model Fit Indices	CFI	0.977
TLI	0.969
RMSEA	0.082
SRMR	0.169

## 4. Conclusion

Pesticide labels, including FAO pictograms and WHO color codes, are expected to guide farmers to limit the impact of hazardous pesticides on the health and the environment [25]. Based on the literature recommendation for a standardized assessment tool of the effectiveness of these pictorial and colored messages [10,11,13] this paper piloted the level of farmers’ understanding of FAO pictograms and WHO color codes and the interrelated practices of pesticide handling among Lebanese farmers. An anticipated output is to provide agricultural professionals and public health scientists with a new reliable tool (PLQ) to assess the global farmers’ understanding of pesticide labels, especially FAO pictograms and WHO color codes. The outcome and implications of this tool may provide a database to estimate the extent of the safe use of pesticides in both developed and developing countries.

The item pool development incorporated 29 items from qualitative findings to expand upon relevant concepts and guide questionnaire construction. Drafting the item pool involved thorough examination of existing literature, assessment tools, the International Code of Conduct on Pesticide Management – FAO Guidelines on Good Labelling Practice for Pesticides, and qualitative interview data. Throughout this phase, the language of the items underwent multiple revisions to ensure clarity and readability.

During the questionnaire development process, items underwent qualitative and quantitative testing in the face validity stage, assessed by farmers and head nurses. Subsequently, in the content validity stage, evaluation was conducted by experts in soft skills and professionals experienced in questionnaire development within the relevant field [32,33].

During the assessment of the questionnaire’s psychometric properties, construct validity was confirmed through exploratory and confirmatory factor analyses. In the study, EFA of the PLQ indicated that all items contributed to the model fit of the component, a conclusion supported by both face and content validity.

The PLQ is a well-validated, reliable, and reasonably comprehensive instrument that assesses farmers’ comprehension of pesticide labels especially FAO pictograms and WHO color codes, in addition to the farmers’ adherence to technical sign-related practices, in both English and Arabic languages. Taken together, the PLQ can be utilized at the levels of comprehension and practices among pesticide users. However, further research is continually necessary to enhance the future refinement of the PLQ, ensuring its relevance and appropriateness for timely use as a questionnaire.

### 4.1. The decision-making step

After conducting rigorous psychometric testing including assessments for face, content, and construct validation, the PLQ has undergone initial psychometric evaluation, including exploratory factor analysis and reliability testing, which suggest promising evidence for its construct validity and internal consistency. The results of these tests have confirmed the questionnaire’s strength and reliability in measuring the farmers’ understanding of the pictograms communicated on pesticides and regular pesticide practices. However, further psychometric assessments are recommended to confirm the robustness of the instrument across various demographic groups and contexts. Future studies should focus on additional analyses, including confirmatory factor analysis and examination of differential item functioning, to enhance the PLQ’s applicability and reliability. This foundational work establishes PLQ as a valuable tool for assessing farmers’ understanding of pesticide labeling, while also highlighting the need for ongoing refinement and validation through field testing and comprehensive psychometric evaluations.

### 4.2. Limitations

Despite the thorough development and validation of the PLQ, several limitations related to the sample, question format, and respondent characteristics contributed to the few weak values obtained throughout the validation steps:

#### 4.2.1. Sample size.

The sample size of 107 respondents, while adequate for initial validation, may not be large enough to fully generalize the findings. According to statistical guidelines, larger sample sizes are recommended to increase the robustness and stability of factor analyses and reliability tests. A sample size of at least 200 participants would be ideal for ensuring a more representative distribution and for enhancing the generalizability of the results to broader populations.

#### 4.2.2. Nature of the questionnaire.

The questionnaire included both binary (dichotomous) and ordinal items, which may have affected the depth of responses. The binary format (Yes/No options) limits the scope of responses, which is particularly challenging when assessing complex behaviors or understanding. Furthermore, the binary items lack a gradation of responses, restricting the ability to detect nuances in respondent knowledge or attitudes. This may have led to oversimplified responses and potentially reduced the precision of the measurement.

#### 4.2.3. Respondent demographics and characteristics.

- **Age Distribution**: The age distribution of the respondents (61% between 40 and 60 years old, and 25% aged above 60) introduces an additional limitation. Older respondents may have different agricultural practices and safety perceptions, influenced by their longer experience but possibly outdated knowledge. These age-related differences in pesticide use and safety awareness could contribute to varying interpretations of the pesticide labels and safety pictograms.
- **Education Level**: The educational background of the respondents also represents a significant limitation. With 21% of participants being illiterate and 61% having only elementary to middle school education, the sample has a low level of formal education. This likely affected the respondents’ ability to comprehend the technical language of the questionnaire, interpret pesticide labels accurately, and understand the environmental and health implications of pesticide use.
- **Lack of Training**: A major concern is that 92% of the respondents reported having never received formal training on pesticide safety. This lack of training may have contributed to gaps in their knowledge, leading to inaccurate or incomplete understanding of the pesticide labels, pictograms, and safety protocols. This deficiency could result in lower reliability of responses, as the farmers’ answers may reflect outdated practices or a general lack of awareness of current standards.

#### 4.2.4. Sample homogeneity and type.

The sample was exclusively composed of farmers, which introduced potential bias due to the homogeneity of the respondent group. While this focus on farmers is appropriate for the study’s context, the lack of diversity in professional background, education level, and exposure to pesticide training limits the generalizability of the findings to other populations, such as agricultural technicians or consumers who may have different levels of knowledge and experience. Furthermore, farmers from different regions or agricultural practices (*e.g.,* crop types or pesticide usage) might exhibit different levels of understanding, which could impact the results.

#### 4.2.5. Potential bias due to limited knowledge and experience.

The combination of illiteracy, low education levels, and the absence of formal pesticide safety training suggests that a significant portion of the sample may not have sufficient prior knowledge of pesticide labels or their associated safety measures. This lack of knowledge could have influenced the accuracy of their responses, particularly on items that required a deeper understanding of modern pesticide safety practices. Additionally, respondents may have been more inclined to rely on traditional practices or local knowledge, which may not always align with current scientific standards or regulatory practices.

#### 4.2.6. Cultural and regional differences.

The study’s sample was limited to a specific group of Lebanese farmers, and while this context is valuable for localizing the findings, it also limits the external validity of the results. Pesticide use practices, awareness, and training can vary widely across regions and cultures, so the findings may not be applicable to farmers in other countries or regions where training, education, and practices differ.

### 4.3. Implications for practice

To guarantee effective and professional farming practices, it is essential to possess a reliable, accurate, and applicable evaluation tool. The availability of a validated assessment instrument that measures the farmers’ understanding of the pictograms communicated on pesticides and the regular pesticide practices in Lebanon is crucial and holds significant importance. This questionnaire functions as an appraisal instrument suitable for ongoing evaluation within the Ministry of Agriculture and non-governmental organizations rehabilitation projects.

## Supporting information

S1 File Appendix. (DOCX)
