# Supplementary material for: Development and validation of the pesticides label (pictograms and color codes) questionnaire: A pilot study of farmers’ understanding and practices in Lebanon
Source: PLoS One. 2025 Jun 9;20(6):e0321591. doi: 10.1371/journal.pone.0321591 (PMC12148187; doi:10.1371/journal.pone.0321591)
Supplement: S1 File — (DOCX) [file pone.0321591.s001.docx]

**Appendix A**

**Section 1: Pictorial and Color Code Comprehension Assessment**

Q1-14: What do these pictorial signs on pesticide labels mean?

Q15-18: What do these color signs on pesticide labels mean?

| **Pictogram** | **Meaning** | **Pictogram** | **Meaning** |
| --- | --- | --- | --- |
| Q1: 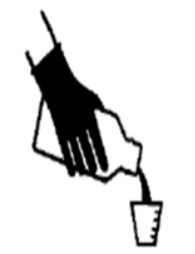 |  | Q2: 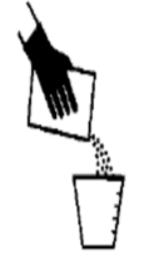 |  |
| Q3: 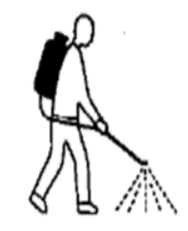 |  | Q4: 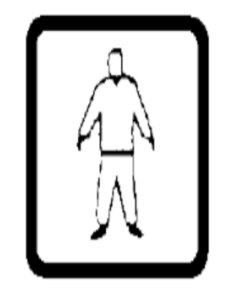 |  |
| Q5: 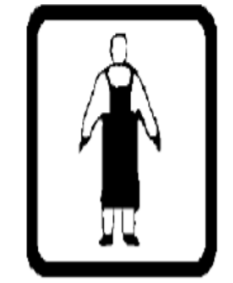 |  | Q6: 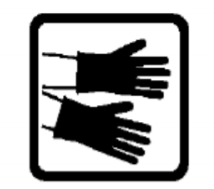 |  |
| Q7: 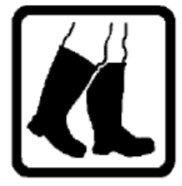 |  | Q8: 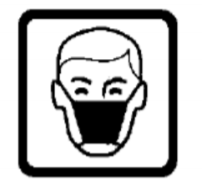 |  |
| Q9: 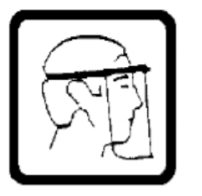 |  | Q10: 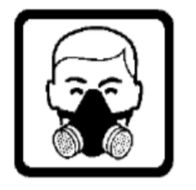 |  |
| Q11: 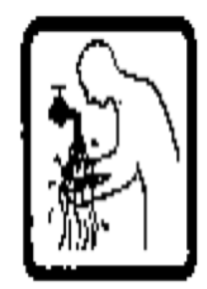 |  | Q12: 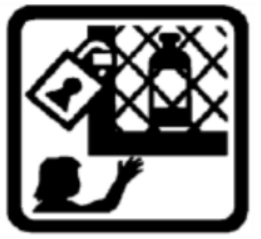 |  |
| Q13: 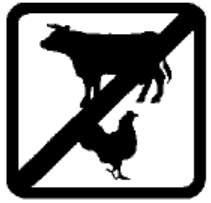 |  | Q14:  |  |
| **Color Code** | **Meaning** | **Color Code** | **Meaning** |
| Q15:  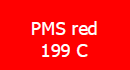 |  | Q16:  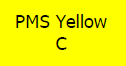 |  |
| Q17:  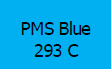 |  | Q18:  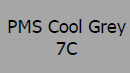 |  |
| **Q19 – What does this sequence of pictorial and color signs on pesticide label mean?**  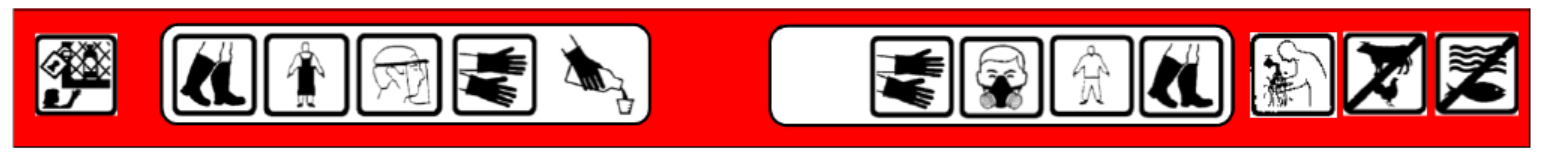 | | | |
| **Meaning:** | | | |

**Understanding level scale:** Each item was assigned 2 scores (correct response), 1 score (partially correct response), and zero score (incorrect response). Then, the mean score was calculated and divided into intervals: weak [0, 12], moderate [13, 24], and good [25-36].

**Section 2: Technical Sign-related Practice Assessment**

| **Practices** | Yes No | If not, why? |
| --- | --- | --- |
| 1. When necessary, I put protection over my nose and mouth during pesticide application. | 🞏 🞏 |  |
| 1. When necessary, I wear protective rubber shoes during pesticide application. | 🞏 🞏 |  |
| 1. When necessary, I wear protective gloves during pesticide application. | 🞏 🞏 |  |
| 1. When necessary, I wear protective clothes during pesticide application (overall or apron). | 🞏 🞏 |  |
| 1. When necessary, I wear eye protection during pesticide application. | 🞏 🞏 |  |
| 1. When necessary, I wear a protective respirator during pesticide application. | 🞏 🞏 |  |
| 1. I wash my hands after pesticide use. | 🞏 🞏 |  |
| 1. I keep pesticide products locked out and out of reach of children. | 🞏 🞏 |  |
| 1. I keep pesticides away from farm animals. | 🞏 🞏 |  |
| 1. I keep pesticides away from water resources (lakes, rivers, ponds, and streams) | 🞏 🞏 |  |

**Practice level scale:** each item was assigned zero score (No) and 1 score (Yes). The mean score of practice was then divided into intervals: weak [0, 4], moderate [5, 8], and good [9-11].

**Appendix B**

The face validity survey invited experts to evaluate whether items in the PLQ are difficult or obscure by rating them on a scale from 1 to 4. The experts were asked as well to rate the level of importance of each item on the same scale as follows:

| Difficulty: How difficult is this item? | | | 1 = Very difficult | |  |
| --- | --- | --- | --- | --- | --- |
|  |  |  | 2 = Difficult | |  |
|  |  |  | 3 = Somewhat difficult | |  |
|  |  |  | 4 = Not difficult | |  |
| Obscurity: Is this item obscure? | | | 1 = Very obscure | |  |
|  |  |  | 2 = Obscure | |  |
|  |  |  | 3 = Somewhat obscure | |  |
|  |  |  | 4 = Not obscure | |  |
| Level of Importance: How important is this item? | | | 1 = Not important | |  |
|  | | | 2 = somewhat important | |  |
|  | | | 3 = quite important | |  |
|  | | | 4 = Very important | |  |
| The table below shows the content validity assessment done by 10 experts on each item of the questionnaire. | | | | |  |
|  | **Face Validity Assessment by 10 Farmers** | | | | |
|  | **Qualitative** | | | **Quantitative** | |
| **Item** | **FVI of Difficulty** | **FVI of Obscurity** | | **FVI of Importance** | |
| 1 | 1 | 1 | | 1 | |
| 2 | 1 | 1 | | 1 | |
| 3 | 1 | 1 | | 1 | |
| 4 | 1 | 1 | | 0,93 | |
| 5 | 1 | 1 | | 0,93 | |
| 6 | 1 | 1 | | 1 | |
| 7 | 0,98 | 0,98 | | 1 | |
| 8 | 1 | 1 | | 0,98 | |
| 9 | 0,95 | 0,95 | | 0,95 | |
| 10 | 0,98 | 0,9 | | 0,98 | |
| 11 | 1 | 1 | | 1 | |
| 12 | 0,95 | 0,95 | | 1 | |
| 13 | 0,95 | 0,93 | | 0,93 | |
| 14 | 0,98 | 0,95 | | 0,95 | |
| 15 | 1 | 1 | | 0,98 | |
| 16 | 0,98 | 1 | | 1 | |
| 17 | 1 | 1 | | 1 | |
| 18 | 1 | 1 | | 1 | |
| 19 | 0,93 | 0,88 | | 0,88 | |
| 20 | 1 | 1 | | 1 | |
| 21 | 1 | 1 | | 1 | |
| 22 | 0,98 | 1 | | 0,98 | |
| 23 | 0,93 | 0,90 | | 1 | |
| 24 | 1 | 1 | | 0,98 | |
| 25 | 0,95 | 1 | | 1 | |
| 26 | 1 | 1 | | 1 | |
| 27 | 1 | 0,98 | | 0,95 | |
| 28 | 1 | 0,98 | | 0,98 | |
| 29 | 1 | 1 | | 1 | |
| **Average** | 0,98 | 0,98 | | 0,98 | |

**Appendix C**

**Part A:**

The content validity survey invited the experts to evaluate whether items in the PLQ are simple, clear, and appropriate based on their location by rating them as follows:

| Simplicity: How simple is this item? | 1 = Not simple |
| --- | --- |
|  | 2 = Somewhat simple |
|  | 3 = Quite simple |
|  | 4 = Very simple |
| Clarity: Is this item clear? | 1 = Not clear 2 = item needs some revision 3 = clear 4 = Very clear |
| Appropriateness: How essential is this item? | 1 = Not Appropriate |
|  | 2 = somewhat appropriate |
|  | 3 = Appropriate |
|  | 4 = Not appropriate |
| Relevance: How relevant is this item? | 1 = Not relevant |
|  | 2 = somewhat relevant |
|  | 3 = quite relevant |
|  | 4 = Very relevant |

The table below shows the content validity assessment done by eight experts on each item of the questionnaire.

|  | **Content Validity Assessment by 8 Experts** | | | | |
| --- | --- | --- | --- | --- | --- |
|  | **Qualitative** | | | **Quantitative** | |
| **Item** | **CVI of Simplicity** | **CVI of Clarity** | **CVI of Appropriateness** | **I-CVI** | **UA** |
| 1 | 1 | 1 | 1 | 1 | 1 |
| 2 | 1 | 1 | 1 | 1 | 1 |
| 3 | 1 | 1 | 1 | 1 | 1 |
| 4 | 1 | 1 | 1 | 1 | 1 |
| 5 | 0,88 | 0,88 | 0,88 | 1 | 1 |
| 6 | 1 | 1 | 0,91 | 1 | 1 |
| 7 | 1 | 0,97 | 1 | 1 | 1 |
| 8 | 1 | 1 | 1 | 1 | 1 |
| 9 | 1 | 1 | 1 | 1 | 1 |
| 10 | 1 | 1 | 0,94 | 1 | 1 |
| 11 | 1 | 1 | 1 | 1 | 1 |
| 12 | 0,88 | 0,88 | 1 | 1 | 1 |
| 13 | 1 | 0,91 | 0,91 | 1 | 1 |
| 14 | 1 | 1 | 1 | 0,88 | 0 |
| 15 | 1 | 1 | 1 | 1 | 1 |
| 16 | 1 | 0,97 | 1 | 1 | 1 |
| 17 | 1 | 1 | 1 | 1 | 1 |
| 18 | 1 | 1 | 1 | 1 | 1 |
| 19 | 0,81 | 0,97 | 0,81 | 0,88 | 0 |
| 20 | 1 | 1 | 1 | 1 | 1 |
| 21 | 1 | 1 | 1 | 1 | 1 |
| 22 | 1 | 0,97 | 1 | 0,88 | 0 |
| 23 | 1 | 0,91 | 0,94 | 1 | 1 |
| 24 | 1 | 0,91 | 1 | 1 | 1 |
| 25 | 0,97 | 0,97 | 0,97 | 1 | 1 |
| 26 | 0,97 | 1 | 1 | 1 | 1 |
| 27 | 1 | 1 | 0,97 | 0,88 | 0 |
| 28 | 1 | 1 | 1 | 1 | 1 |
| 29 | 1 | 1 | 0,91 | 1 | 1 |
| **Average** | **0,98** | 0,98 | 0,97 | 0,98 | S-CVI/Ave |

**Part B:**

The content validity survey invited the experts to evaluate whether items in the PLQ are essential by rating them as follows:

|  | Expert 1 | Expert 2 | Expert 3 | Expert 4 | Expert 5 | Expert 6 | Expert 7 | Expert 8 | CVR | |  |
| --- | --- | --- | --- | --- | --- | --- | --- | --- | --- | --- | --- |
| Item 1 | x | x | x | x | x | x | x | x | 1 | |  |
| Item 2 | x | x | x | x | x | x | x | x | 1 | |  |
| Item 3 | x | x | x | x | x | x | x | x | 1 | |  |
| Item 4 | x | x | x | x | x | x | x | x | 1 | |  |
| Item 5 | x | x | x |  | x | x | x | x | 0,75 | |  |
| Item 6 | x | x | x | x | x | x | x | x | 1 | |  |
| Item 7 | x | x | x | x | x | x | x | x | 1 | |  |
| Item 8 | x | x | x | x | x | x | x | x | 1 | |  |
| Item 9 | x | x | x | x | x | x | x | x | 1 | |  |
| Item 10 | x | x | x | x | x | x | x | x | 1 | |  |
| Item 11 | x | x | x | x | x | x | x | x | 1 | |  |
| Item 12 | x | x | x | x | x | x | x | x | 1 | |  |
| Item 13 | x | x | x | x | x | x | x | x | 1 | |  |
| Item 14 | x | x | x | x | x | x | x | x | 1 | |  |
| Item 15 | x | x | x | x | x | x | x | x | 1 | |  |
| Item 16 | x | x | x | x | x | x | x | x | 1 | |  |
| Item 17 | x | x | x | x | x | x | x | x | 1 | |  |
| Item 18 | x | x | x | x | x | x | x | x | 1 | |  |
| Item 19 | x |  | x | x | x | x | x | x | 0,75 | |  |
| Item 20 | x | x | x | x | x | x | x | x | 1 | |  |
| Item 21 | x | x | x | x | x | x | x | x | 1 | |  |
| Item 22 | x | x | x | x | x | x | x | x | 1 | |  |
| Item 23 | x | x | x | x | x | x | x | x | 1 | |  |
| Item 24 | x | x | x | x | x | x | x | x | 1 | |  |
| Item 25 |  | x | x | x | x | x | x | x | 0,75 | |  |
| Item 26 | x | x | x | x | x | x | x | x | 1 | |  |
| Item 27 | x | x | x | x | x | x | x | x | 1 | |  |
| Item 28 | x | x | x | x | x | x | x | x | 1 | |  |
| Item 29 | x | x | x | x | x |  | x | x | 0,75 | |  |
| CVR(Critical) for a panel size (N) of 8 is 0,75. | | | | | | | | | | 0,97 | |

**Appendix D (Tetrachoric Correlation for Section I)**


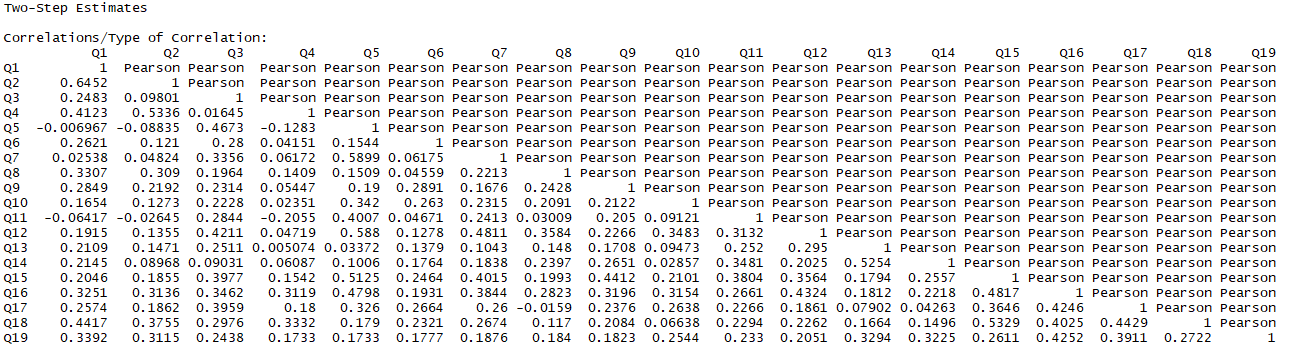

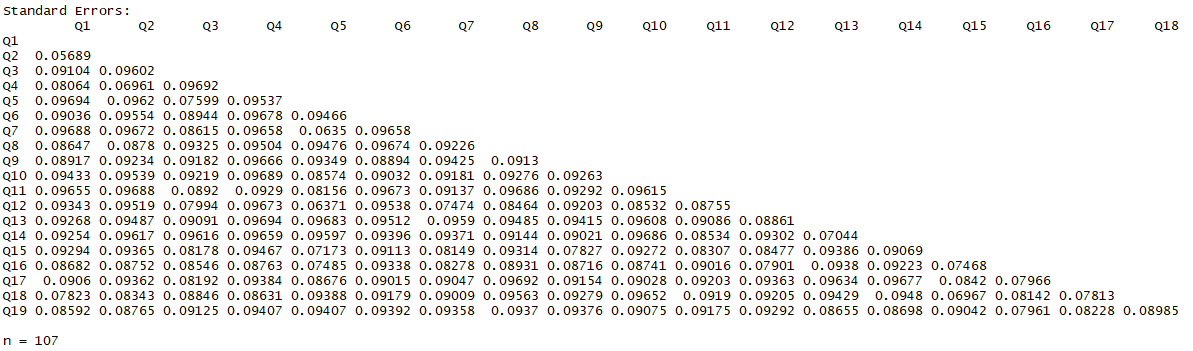


**Appendix E (Tetrachoric Correlation for Section II)**


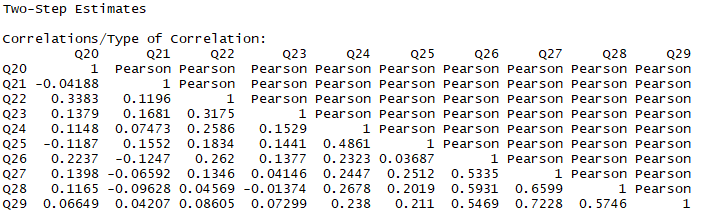


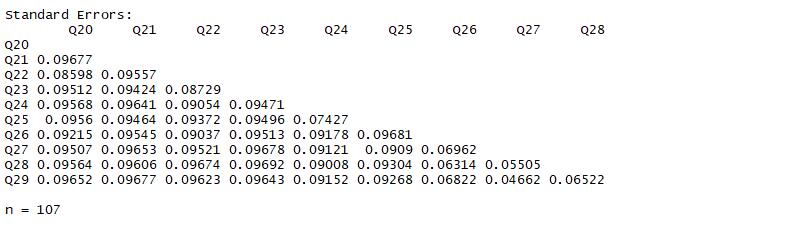


**Appendix F – CFA for Section I**

| **Latent Variables** | | | | | | |
| --- | --- | --- | --- | --- | --- | --- |
| **factor1** | **Estimate** | **Std.Err** | **z-value** | **P(>\|z\|)** | **Std.lv** | **Std.all** |
| **Q1** | 1 |  |  |  | 0,416 | 0,496 |
| **Q2** | 0,843 | 0,114 | 7,417 | 0 | 0,351 | 0,425 |
| **Q3** | 0,18 | 0,18 | 0,561 | 0 | 0,218 | 0,259 |
| **Q4** | 0,169 | 0,15 | 1,133 | 0 | 0,218 | 0,277 |
| **Q5** | 0,524 | 0,16 | 3,642 | 0 | 0,259 | 0,626 |
| **Q6** | 0,96 | 0,193 | 4,966 | 0 | 0,31 | 0,676 |
| **Q7** | 0,936 | 0,179 | 5,233 | 0 | 0,353 | 0,671 |
| **Q8** | 0,849 | 0,165 | 5,146 | 0 | 0,359 | 0,671 |
| **factor2** | **Estimate** | **Std.Err** | **z-value** | **P(>\|z\|)** | **Std.lv** | **Std.all** |
| **Q11** | 1 |  |  |  | 0,288 | 0,412 |
| **Q12** | 1,178 | 0,241 | **4,891** | 0 | 0,363 | 0,638 |
| **Q13** | 0,867 | 0,226 | 3,836 | 0 | 0,249 | 0,376 |
| **Q14** | 2,067 | 0,395 | 5,223 | 0 | 0,595 | 0,698 |
| **Q15** | 1,884 | 0,392 | 4,804 | 0 | 0,542 | 0,707 |
| **Q16** | 1,781 | 0,36 | 4,952 | 0 | 0,359 | 0,515 |
| **Q17** | 1,07 | 0,379 | 4,627 | 0 | 0,509 | 0,575 |
| **Q18** | 0,28 | 0,183 | 1,529 | 0,127 | 0,308 | 0,515 |
| **Covariances** | | | | | | |
|  | **Estimate** | **Std.Err** | **z-value** | **P(>\|z\|)** | **Std.lv** | **Std.all** |
| **factor1** | 0,115 | 0,028 | 4,055 | 0 | 0,962 | 0,962 |
| **Variances** | | | | | | |
|  | **Estimate** | **Std.Err** | **z-value** | **P(>\|z\|)** | **Std.lv** | **Std.all** |
| **.Q1** | 0,532 | 0,053 | 9,964 | 0 | 0,532 | 0,754 |
| **.Q2** | 0,558 | 0,056 | 9,956 | 0 | 0,558 | 0,819 |
| **.Q3** | 0,476 | 0,073 | 6,05 | 0 | 0,476 | 0,62 |
| **.Q4** | 0,659 | 0,164 | 12,555 | 0 | 0,659 | 0,933 |
| **.Q5** | 0,497 | 0,07 | 7,058 | 0 | 0,497 | 0,608 |
| **.Q6** | 0,479 | 0,149 | 3,208 | 0,001 | 0,479 | 0,688 |
| **.Q7** | 0,365 | 0,043 | 8,424 | 0 | 0,365 | 0,696 |
| **.Q8** | 0,536 | 0,068 | 9,89 | 0 | 0,536 | 0,778 |
| **.Q9** | 0,616 | 0,134 | 10,5 | 0 | 0,616 | 0,834 |
| **.Q10** | 0,405 | 0,054 | 7,44 | 0 | 0,405 | 0,83 |
| **.Q11** | 0,378 | 0,057 | 6,617 | 0 | 0,378 | 0,859 |
| **.Q12** | 0,373 | 0,045 | 8,245 | 0 | 0,373 | 0,793 |
| **.Q13** | 0,658 | 0,062 | 10,577 | 0 | 0,658 | 0,799 |
| **.Q14** | 0,68 | 0,13 | 5,25 | 0 | 0,68 | 0,758 |
| **.Q15** | 0,623 | 0,055 | 11,272 | 0 | 0,623 | 0,682 |
| **.Q16** | 0,658 | 0,063 | 10,43 | 0 | 0,658 | 0,672 |
| **.Q17** | 0,4 | 0,054 | 7,41 | 0 | 0,4 | 0,809 |
| **.Q18** | 0,625 | 0,05 | 12,5 | 0 | 0,625 | 0,854 |
| **factor1** | 0,173 | 0,031 | 5,555 | 0 | 1 | 1 |
| **factor2** | 0,083 | 0,032 | 2,704 | 0,007 | 1 | 1 |

**Appendix G – CFA for Section II**

| **Latent Variables** | | | | | | |
| --- | --- | --- | --- | --- | --- | --- |
|  | **Estimate** | **std.Err** | **z-value** | **p(>\|zl)** | **std.lv** | **std.all** |
| **factor1 =** |  |  |  |  |  |  |
| **Q20** | 1.000 |  |  |  | 0.401 | 0.401 |
| **Q21** | 0.233 | 0.427 | 0.545 | 0.586 | 0.093 | 0.093 |
| **Q22** | 1.564 | 0.519 | 3.013 | 0.003 | 0.627 | 0.627 |
| **Q23** | 1.190 | 0.531 | 2.242 | 0.025 | 0.477 | 0.477 |
| **Q24** | 2.278 | 0.941 | 2.420 | 0.016 | 0.913 | 0.913 |
| **factor2 =** |  |  |  |  |  |  |
| **Q25** | 1.000 |  |  |  | 0.682 | 0.682 |
| **Q26** | 1.268 | 0.224 | 5.660 | 0.000 | 0.865 | 0.865 |
| **Q27** | 1.427 | 0.251 | 5.679 | 0.000 | 0.973 | 0.973 |
| **Q28** | 1.338 | 0.240 | 5.569 | 0.000 | 0.913 | 0.913 |
| **Q29** | 1.407 | 0.249 | 5.646 | 0.000 | 0.960 | 0.960 |
| **Covariances** | | | | | | |
|  | **Estimate** | **std.Err** | **z-value** | **p(>\|z\|)** | **std.lv** | **std.all** |
| **factor1 -** |  |  |  |  |  |  |
| **factor2** | 0.189 | 0.084 | 2.248 | 0.025 | 0.691 | 0.691 |
| **Thresholds** | | | | | | |
|  | **Estimate** | **std.Err** | **z-value** | **p(>\|z\|)** | **std.lv** | **std.all** |
| **Q20 t1** | -0.610 | 0.130 | -4.678 | 0.000 | -0.610 | -0.610 |
| **Q21 t1** | 0.129 | 0.122 | 1.058 | 0.290 | 0.129 | 0.129 |
| **Q22 t1** | -0.371 | 0.125 | -2.976 | 0.003 | -0.371 | -0.371 |
| **Q23 t1** | 0.727 | 0.134 | 5.418 | 0.000 | 0.727 | 0.727 |
| **Q24 t1** | -0.106 | 0.122 | -0.866 | 0.387 | -0.106 | -0.106 |
| **Q25 t1** | 0.371 | 0.125 | 2.976 | 0.003 | 0.371 | 0.371 |
| **Q26 t1** | -1.168 | 0.157 | -7.425 | 0.000 | -1.168 | -1.168 |
| **Q27 t1** | -1.266 | 0.165 | -7.684 | 0.000 | -1.266 | -1.266 |
| **Q28 t1** | -1.215 | 0.161 | -7.559 | 0.000 | -1.215 | -1.215 |
| **Q29 t1** | -1.441 | 0.181 | -7.967 | 0.000 | -1.441 | -1.441 |
| **Variances** | | | | | | |
|  | **Estimate** | **std.Err** | **z-value** | **p(>\|z\|)** | **std.lv** | **std. all** |
| **Q20** | 0.839 |  |  |  | 0.839 | 0.839 |
| **.Q21** | 0.991 |  |  |  | 0.991 | 0.991 |
| **.Q22** | 0.607 |  |  |  | 0.607 | 0.607 |
| **Q23** | 0.772 |  |  |  | 0.772 | 0.772 |
| **.Q24** | 0.166 |  |  |  | 0.166 | 0.166 |
| **Q25** | 0.534 |  |  |  | 0.534 | 0.534 |
| **Q26** | 0.251 |  |  |  | 0.251 | 0.251 |
| **.Q27** | 0.053 |  |  |  | 0.053 | 0.053 |
| **.Q28** | 0.166 |  |  |  | 0.166 | 0.166 |
| **Q29** | 0.079 |  |  |  | 0.079 | 0.079 |
| **factor1** | 0.161 | 0.113 | 1.419 | 0.156 | 1.000 | 1.000 |
| **factor2** | 0.466 | 0.156 | 2.993 | 0.003 | 1.000 | 1.000 |
